# Synthesis and Characterization of 4-Indolylcyanamide: A Potential IR Probe for Local Environment

**DOI:** 10.3390/molecules30204063

**Published:** 2025-10-12

**Authors:** Min You, Qingxue Li, Zilin Gao, Changyuan Guo, Liang Zhou

**Affiliations:** 1 School of Computer Science and Engineering, Chongqing Three Gorges University, Chongqing 404100, China; minyou@sanxiau.edu.cn (M.Y.); zilingao@sanxiau.edu.cn (Z.G.); 2 School of Physics and Astronomy, Applied Optics Beijing Area Major Laboratory, Center for Advanced Quantum Studies, Beijing Normal University, Beijing 100875, China; liqingxue22@mails.ucas.ac.cn; 3 Laboratory of Soft Matter Physics, Institute of Physics, Chinese Academy of Sciences, Beijing 100190, China; 4 Key Laboratory of Intelligent Air-Ground Cooperative Control for Universities in Chongqing, College of Automation, Chongqing University of Posts and Telecommunications, Chongqing 400065, China; woodenguo@nuaa.edu.cn; 5 State Key Laboratory of Thorium Energy, Shanghai Institute of Applied Physics, Chinese Academy of Sciences, Shanghai 201800, China

**Keywords:** indole derivative, infrared probe, cyanamide group, vibrational characteristics, FTIR, pump–probe spectroscopy, quantum chemical calculations

## Abstract

This study reports the synthesis and comprehensive spectroscopic characterization of 4-indolylcyanamide (4ICA), a novel indole-derived infrared (IR) probe designed for assessing local microenvironments in biological systems. 4ICA was synthesized via a two-step procedure with an overall yield of 43%, and its structure was confirmed using high-resolution mass spectrometry and ^1^HNMR. Fourier Transform Infrared (FTIR) spectroscopy revealed that the cyanamide group stretching vibration of 4ICA exhibits exceptional solvent-dependent frequency shifts, significantly greater than those of conventional cyanoindole probes. A strong linear correlation was observed between the vibrational frequency and the combined Kamlet–Taft parameter, underscoring the dominant role of solvent polarizability and hydrogen bond acceptance in modulating its spectroscopic behavior. Quantum chemical calculations employing density functional theory (DFT) with a conductor-like polarizable continuum model (CPCM) provided further insight into the solvatochromic shifts and suppression of Fermi resonance in high-polarity solvents such as DMSO. Additionally, IR pump–probe measurements revealed short vibrational lifetimes (~1.35 ps in DMSO and ~1.13 ps in ethanol), indicative of efficient energy relaxation. With a transition dipole moment nearly twice that of traditional nitrile-based probes, 4ICA demonstrates enhanced sensitivity and signal intensity, establishing its potential as a powerful tool for site-specific environmental mapping in proteins and complex biological assemblies using nonlinear IR techniques.

## 1. Introduction

Indole derivatives have attracted substantial attention in biochemistry and chemical biology owing to their structural versatility and diverse biological functions. These compounds exhibit a wide spectrum of pharmacological activities, including anti-proliferative [1,2], antibacterial [3,4], anti-inflammatory activity [5], antiviral [6,7,8], antidiabetic [9,10], anticonvulsant [11], antihypertension [12], Alzheimer’s disease [13], antimalarial [14] and other miscellaneous diseases. Their broad utility arises from the indole scaffold’s ability to engage in hydrogen bonding [15,16], π–π stacking [17,18], and electrostatic interactions [19,20], rendering it a privileged structure in drug discovery and molecular probe design.

A prominent natural indole derivative is the essential amino acid tryptophan (Trp), which plays a vital role in protein structure, dynamics, and function. Modifications of the Trp indole ring with spectroscopic reporter groups have proven particularly valuable for probing biomolecular environments. In this context, cyano-substituted tryptophans (cyanotryptophans) have emerged as sensitive site-specific probes in fluorescence [21,22,23,24] or vibrational spectroscopy [25,26,27]. Their nitrile group (CN) exhibits a stretching vibration in a spectrally transparent region (≈2100–2300 cm^−1^) of the IR spectrum, enabling the detection of subtle environmental changes such as solvent polarity, hydrogen bonding, and local electrostatics [25,27,28]. Among cyanotryptophans, 5-cyanoindole has been extensively employed as an IR probe, owing to the well-defined linear correlation between its nitrile stretching frequency and solvent polarity parameters [28]. Similarly, 4-cyanoindole (4CI) has gained attention for its unique Fermi resonance properties, which make it particularly effective in assessing hydration status and local solvation dynamics [27]. Incorporation of cyanoindole derivatives into proteins—for instance, 5-cyanoindole-substituted Trp in the influenza A M2 proton channel—has provided critical insights into hydrogen-bonding interactions at functional gating sites [26].

Despite these advances, the broader application of cyanotryptophans in nonlinear IR spectroscopies such as two-dimensional IR (2D-IR) spectroscopy remains limited. The challenges primarily stem from their relatively weak transition dipole moments, which generate modest signal intensities, compounded by the typically low concentrations achievable in biological samples. To address these limitations, ongoing research has focused on designing novel indole-based vibrational reporters with enhanced spectroscopic properties. For example, introducing electron-withdrawing groups such as nitriles, or esters onto the indole ring has been shown to modulate transition dipole strengths, tune vibrational lifetimes, and improve probe sensitivity [25,29]. It is important to note that the isonitrile group (NC) features a divalent carbon atom bonded to nitrogen via a formal double bond, resulting in the connectivity R−N^+^≡C^−^, which is distinct from the nitrile group’s R−C≡N connectivity. Thus, recent studies have further explored isonitrile modifications of indole scaffolds as strategies to expand the repertoire of effective IR probes [30,31].

**Figure 1:**
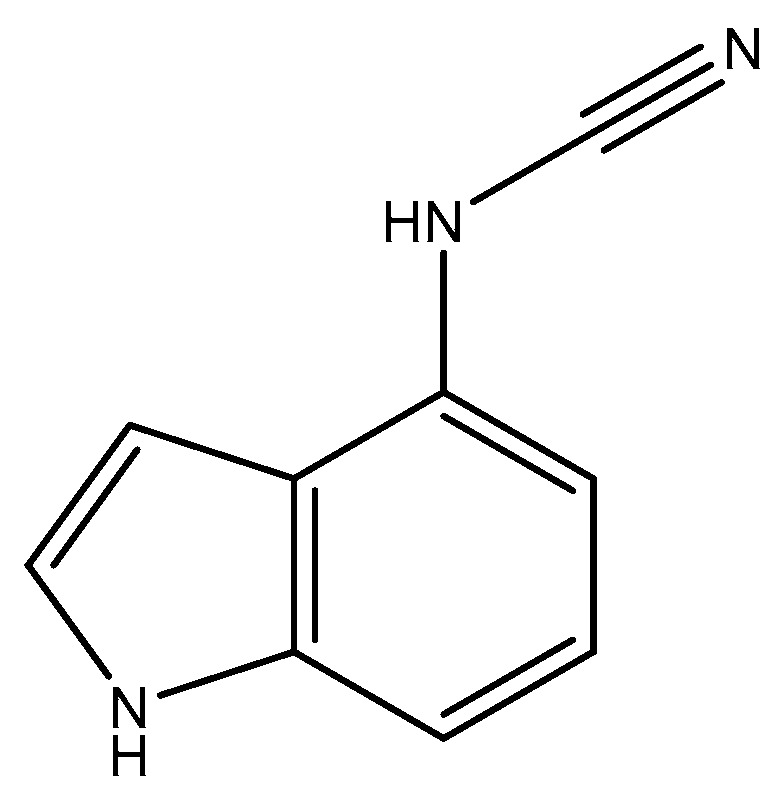
Structure of the 4-indolylcyanamide (4ICA).

This work reports the synthesis of 4-indolylcyanamide (4ICA, Figure 1) and its evaluation as an infrared (IR) probe across diverse solvents. Fourier Transform Infrared (FTIR) spectroscopy was employed to characterize the vibrational behavior of 4ICA across different solvent environments. The influence of solvent properties on the nitrile stretching vibration of cyanamide group (NHCN) was analyzed using the well-established Kamlet–Taft empirical parameters [32,33]. In addition, complementary solvent descriptors such as the Kirkwood–Bauer–Magat (KBM) solvation parameter, defined as *f* = (ε − 1)/(2ε + 1), were also considered [34].

To further elucidate the sensitivity of the NHCN stretching frequency to solvent effects, theoretical calculations were performed for 4ICA in selected solvent systems. Moreover, polarization-controlled IR pump–probe spectroscopy was conducted in representative solvents—dimethyl sulfoxide (DMSO) and ethanol (EtOH)—to investigate the dynamical properties of 4ICA. For comparison, additional spectral data on the nitrile vibration of 4CI are included in the Supplementary Information (Table S1).

## 2. Results and Discussion

### 2.1. Chemistry

**Scheme 1:**
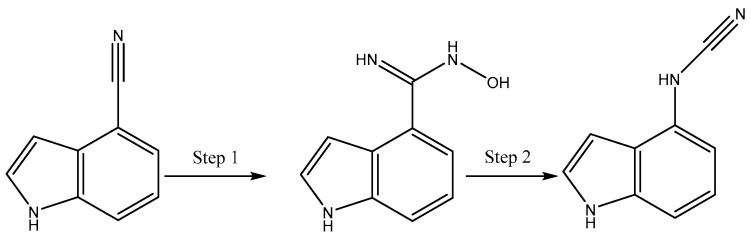
The synthetic pathway of 4ICA.

The title compound, 4ICA, was synthesized as a white powder with an overall yield of 43% over a two-step sequence, as depicted in Scheme 1. 

Step 1: Under an inert nitrogen atmosphere, 4CI (2.5 g) was charged into a three-necked flask and dissolved in anhydrous ethanol (50 mL). A 50% aqueous hydroxylamine solution (2.5 mL, 2.0 equiv) was then added dropwise. The reaction mixture was heated to reflux and maintained at this temperature for 8 h. Upon cooling to ambient temperature, the mixture was concentrated under reduced pressure to afford the crude product (3.0 g), which was utilized directly in the subsequent step without further purification.

Step 2: The crude product from Step 1 was dissolved in anhydrous dichloromethane (DCM, 50 mL) under a nitrogen atmosphere. *N*,*N*-Diisopropylethylamine (DIEA, 2.4 g, 1.05 equiv) was introduced, followed by the dropwise addition of tosyl chloride (TsCl, 3.3 g, 1.0 equiv) in one portion. The reaction was initially stirred at 0 °C (using an ice bath) for 0.5 h, then gradually warmed to room temperature and stirred for an additional 3 h. After reaction completion, the solvent was removed under reduced pressure. The resulting residue was purified via column chromatography using a petroleum ether/ethyl acetate mixture (PE/EA = 2:1 *v*/*v*) as the eluent, yielding the final product (1.2 g).

High-resolution mass spectrometry (HRMS) in electrospray ionization mode (ES) confirmed the molecular ion at *m*/*z* 157.0646 [(M + H)^+^; calculated for C_9_H_7_N_3_: 157.0645]. ^1^H NMR analysis (500 MHz, DMSO-*d*_6_) revealed characteristic signals at δ 11.27 (s, ^1^H, NH), 9.99 (d, *J* = 1.8 Hz, ^1^H), 7.10 (dt, *J* = 2.0, 0.9 Hz, ^1^H), 7.06 (dd, *J* = 8.2, 1.2 Hz, ^1^H), and 6.64 (d, *J* = 1.0 Hz, ^1^H) (Figure S1). Additional characterization data, including HPLC and mass spectrometry results, are presented in Figures S2 and S3.

### 2.2. FTIR Spectroscopy

**Figure 2:**
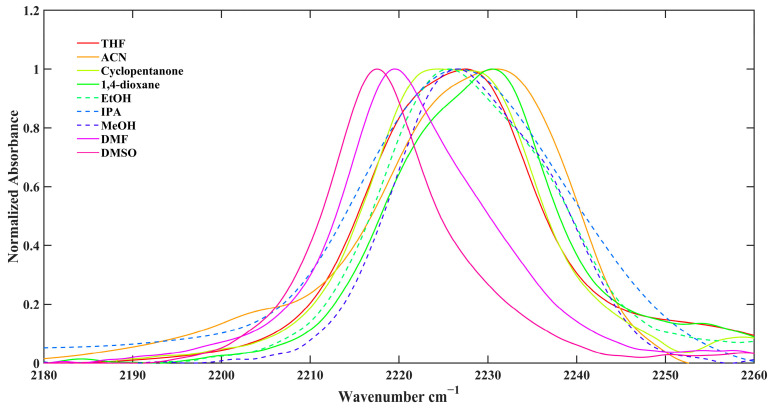
The NHCN stretching vibration of 4ICA in selective solvents.

A systematic investigation of the NHCN stretching vibration of 4-indolylcyanamide (4ICA) was carried out in a selection of pure solvents at room temperature (22 °C), as illustrated in Figure 2. To accurately characterize the spectral features, the full width at half maximum (FWHM) of the NHCN stretching band in each solvent was rigorously extracted through spectral deconvolution employing a Voigt function profile. This approach yielded an excellent fit to the experimental data across all solvent systems examined. The band center, denoted as ω_(NHCN)_, was directly obtained from the absorption maximum in each solvent environment. The detailed numerical results are compiled in Table 1.

Significant spectral differences were observed between polar protic and aprotic solvents. In aprotic solvents such as DMSO, DMF, tetrahydrofuran (THF), 1,4-dioxane, cyclopentanone, and acetonitrile (ACN), a clear trend is observed: the higher the solvent’s β value, the lower the ω_(NHCN)_ vibrational frequency (i.e., a shift to lower wavenumbers, referred to as a red shift). For instance, DMSO with the highest β value (β = 0.76) corresponds to the lowest ω_(NHCN)_ (2217.5 cm^−1^), while solvents with lower β values, such as ACN (β = 0.31) and 1,4-dioxane (β = 0.37), exhibit higher ω_(NHCN)_ values (approximately 2231 cm^−1^). This trend indicates that solvent molecules acting as hydrogen bond acceptors form hydrogen bonds with 4ICA, weakening the force constant of the NHCN bond and thereby reducing its stretching vibrational frequency.

For protic solvents such as methanol (MeOH), ethanol (EtOH), and isopropanol (IPA), the ω_(NHCN)_ values also fall within a relatively low range (2224.5–2226.0 cm^−1^). This observation may be attributed to the collective effect of intricate hydrogen-bonding networks between the solute and solvent molecules, resulting in a less straightforward trend compared to that observed in aprotic solvents.

There is no simple and consistent linear relationship between the solvent polarity parameter π* and ω_(NHCN)_. For instance, *N*,*N*-dimethylformamide (DMF, π* = 0.88) and cyclopentanone (π* = 0.76) have relatively close π* values, yet their ω_(NHCN)_ values differ by 7 cm^−1^ (2219.5 vs. 2226.5 cm^−1^); similarly, EtOH (π* = 0.54) and THF (π* = 0.58) also have comparable π* values, while their ω_(NHCN)_ values differ by 3 cm^−1^ (2225.5 vs. 2228.5 cm^−1^). This indicates that the effect of solvent polarity is likely subordinate to the hydrogen bonding effect with respect to the NHCN stretching vibration.

The full width at half maximum (FWHM) reflects the efficiency of the vibrational relaxation process and is closely related to the solvation environment of the solute and its dynamic changes. The broadening of FWHM is generally associated with stronger solvent–solute interactions, particularly the formation of hydrogen bonds. In protic solvents possessing hydrogen bond donor capability (α > 0), such as IPA, EtOH, and MeOH, the FWHM of the NHCN stretching vibration band is generally broader (20.5–26.7 cm^−1^). Particularly noteworthy is IPA; although it has relatively low polarity, its comparatively high α value (0.76) and β value (0.95) result in the broadest peak. This is likely because the solvent molecules, acting as hydrogen bond donors, engage in dynamic and complex interactions with potential acceptor atoms in the 4ICA molecule. These interactions increase the diversity of solvation structures or accelerate the vibrational energy decay, thereby leading to spectral broadening. In aprotic solvents, the FWHM is relatively narrower (14.0–21.2 cm^−1^). Especially for DMSO, which exhibits the narrowest peak width (14.0 cm^−1^), it indicates a relatively uniform microenvironment for the 4ICA molecules and stable interactions in this type of solvent.

Notably, the NHCN stretching frequency (ω_(NHCN)_) of 4ICA exhibited a larger shift (Δ*ω* ≈ 14 cm^−1^) when transitioning from DMSO to ACN, starkly contrasting with the minimal shift observed for the CN stretching mode of 4CI (Δ*ω* ≈ 3 cm^−1^) under identical solvent conditions (Table S1). This magnitude of shift for 4ICA significantly exceeds that reported for other indole derivatives [25,27,30,31], indicating that the NHCN stretching vibration in 4ICA possesses enhanced sensitivity to solvents compared to conventional indole-based probes.

**Figure 3:**
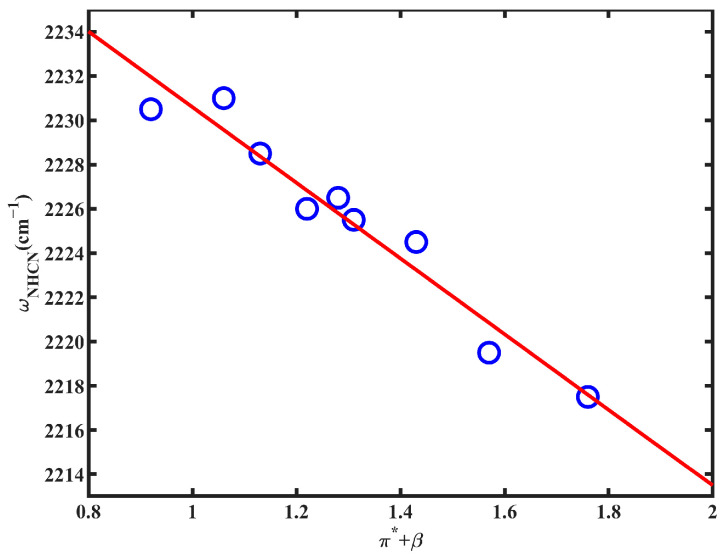
The NHCN vibrational frequency (*ω*_(NHCN)_) of 4ICA as a function of the combined solvent parameter (π* + β). The experimental data (blue circles) from various solvents are fitted by a linear regression (red line).

To quantitatively assess the solvent influence on the NHCN stretching mode—particularly in light of the previously noted roles of hydrogen bond acceptance and solvent polarizability—we performed a linear regression analysis between ω_(NHCN)_ and the combined Kamlet–Taft parameter π* + β. This analysis revealed a strong linear correlation (*R*^2^ = 0.943) across the solvent series (Figure 3), indicating that the vibrational frequency is predominantly governed by these two solvent descriptors. A similar relationship has been reported for 5-cyanoindole [25]. By contrast, no significant correlations were observed between ω_(NHCN)_ and other solvent parameters—such as π*, β, or ε alone, or the FWHM of the NHCN band, or other combined Kamlet–Taft parameters (Figures S4–S6). This linear relationship provides a firm indication that both specific interactions laying a quantitative framework for applying 4ICA to investigate the microscopic environment of proteins in a site-specific manner.

**Figure 4:**
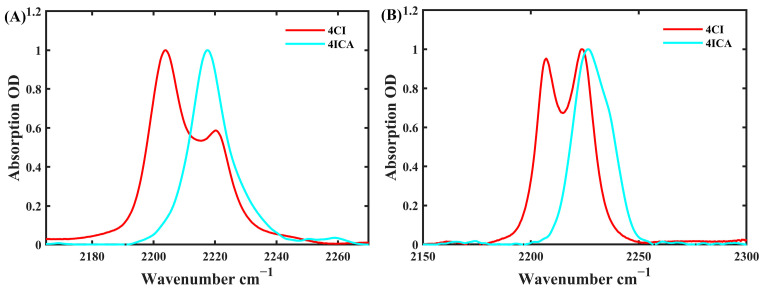
(**A**) Comparison between *ω*_(NHCN)_ of 4ICA and *ω*_(CN)_ of 4CI in pure DMSO; (**B**) Comparison between *ω*_(NHCN)_ of 4ICA and *ω*_(CN)_ of 4CI in pure MeOH solvent.

A comparative analysis of the nitrile stretching vibrations in 4ICA and 4CI, despite their structural analogy, reveals profound spectroscopic distinctions, as illustrated in Figure 4. The CN stretching band of 4CI is characterized by well-resolved doublet features across all solvent environments, a signature attributed to Fermi resonance. In contrast, the corresponding NHCN stretching band of 4ICA demonstrates a significantly attenuated doublet character. The narrowest FWHM = 14.0 cm^−1^ for 4ICA is observed in DMSO (Table 1), indicative of a highly homogeneous solvation environment. This spectral narrowing suggests a potential suppression or masking of the Fermi resonance effect in this specific solvent. Conversely, broader linewidths are recorded in THF (FWHM = 20.2 cm^−1^) and ACN (FWHM = 21.2 cm^−1^). Beyond the influence of solvent polarity, the enhanced broadening in these aprotic solvents may be partially ascribed to Fermi resonance. This is particularly plausible given the extensive electron delocalization within the aromatic ring system of 4ICA, which could promote vibrational coupling between the fundamental NHCN stretch and overtone or combination bands. In protic solvents such as MeOH, hydrogen-bonding interactions induce further spectral broadening (FWHM up to 20.5 cm^−1^), which likely overwhelms and obscures any underlying contribution from Fermi resonance. Furthermore, the nitrile stretching bands of 4CI exhibit a pronounced solvent-dependent intensity pattern. The relative intensity of the lower-frequency peak (near 2215 cm^−1^) is significantly enhanced in protic solvents compared to aprotic environments. This intensity redistribution robustly supports the assignment wherein the lower-frequency peak corresponds to the fundamental CN stretching mode, while the higher-frequency peak (near 2230 cm^−1^) originates from a Fermi resonance interaction—an interpretation consistent with prior reports by Gai et al. [27]. The fundamental origin of the differential Fermi resonance phenomena between 4ICA and 4CI will be explored in the subsequent section. In summary, the presence and modulation of Fermi resonance in 4ICA provide an additional layer of spectral specificity. This enhances its utility as a sensitive IR probe, as its spectral signature becomes uniquely indicative of its local binding state and microenvironment.

The most distinctive property of 4ICA is the exceptionally large transition dipole moment (*µ*^2^) of its NHCN group. Quantitative measurements reveal *µ*^2^ values of 242.5 D^2^ in DMSO and 205.0 D^2^ in ethanol. In contrast, the CN and NC groups exhibit significantly lower *µ*^2^ values, as depicted in Table 2. Thus, the NHCN group’s µ^2^ exceeds those of CN and NC by ~93–135% in DMSO and ~140–164% in ethanol, while CN and NC exhibit comparable magnitudes. Given that CN/NC groups in indole derivatives are established site-specific infrared probes [25,27,30,31], such 2-fold enhancement in *µ*^2^ positions 4ICA as a superior probe for nonlinear IR spectroscopy. This enhancement enables protein studies at substantially lower concentrations while maintaining signal fidelity.

### 2.3. Quantum Chemical Calculations

To elucidate the molecular mechanisms underlying the solvent-dependent shifts in the ω_(NHCN)_ of 4ICA, we performed systematic theoretical calculations. These analyses aimed to identify key factors influencing vibrational behavior across diverse solvent environments. Density functional theory (DFT) calculations were employed to optimize 4ICA structures in both the gas phase and implicit solvent models. Specifically, the conductor-like polarizable continuum model (CPCM) was utilized to simulate solvation effects without explicit solvent ligands. Key computed properties include solvation energies (*E*ₛₒₗᵥₐₜᵢₒₙ, zero-point corrected), dipole moments (*μ*, in Debye), calculated vibrational frequencies corrected by a scaling factor of 0.96 (*ω*_calc_, in cm^−1^), experimental frequencies (*ω*ₑₓₚ, in cm^−1^), the deviation between calculated and experimental values (Δ*ω*, in cm^−1^), C≡N bond lengths (*L*_CN_, in Å), the KBM scaling factor (*f*_KBM_), and dielectric constant (*ε*), all of which are summarized in Table 3. Gas-phase predictions served as the reference state, as experimental gas-phase spectra for 4ICA remain unreported in the literature.

The computed properties of 4ICA in implicit solvent environments, as detailed in Table 3, reveal profound solvent-dependent behaviors rooted in the interplay between solvation dynamics and molecular vibrations. The solvation energy (*E*_solvation_) becomes increasingly negative with rising dielectric constant (*ε*), ranging from −21.2248 kJ/mol in low-polarity 1,4-dioxane (*ε* = 2.2) to −41.3466 kJ/mol in highly polar DMSO (*ε* = 47.2). This trend underscores the enhanced stabilization of 4ICA in polar solvents, where dielectric screening mitigates electrostatic interactions between the solute and solvent. The dipole moment (μ) similarly escalates with solvent polarity, increasing from 4.7969 D in vacuum to 6.4699 D in DMSO. The change in dipole moment (*Δμ*) correlates linearly with *f*_KBM_, reflecting the polarization of the C≡N bond under the influence of solvent electric fields (Figure S6). Such polarization alters the electron density distribution, directly impacting vibrational properties.

The NHCN stretching frequency (*ω*_calc_) exhibits a systematic redshift as solvent polarity increases, shifting from 2237.6 cm^−1^ in 1,4-dioxane to 2225.8 cm^−1^ in DMSO. This redshift arises from the reduction in the vibrational force constant due to solvent-induced electron delocalization. The deviation between calculated and experimental frequencies (Δω) is minimal in protic solvents like methanol (Δ*ω* = −0.13 cm^−1^) and ethanol (Δ*ω* = −0.95 cm^−1^), suggesting that hydrogen bonding partially counteracts dielectric effects. In contrast, aprotic solvents such as DMSO and DMF show larger deviations (−8.31 cm^−1^ and −6.50 cm^−1^, respectively), highlighting the role of specific solvent–solute interactions. The C≡N bond length (*L_CN_*) elongates marginally in polar environments (e.g., Δ*L*_CN_ = 0.00201 Å in DMSO), consistent with reduced bond order from solvation-driven electron redistribution. The KBM scaling factor (*f*_KBM_) decreases with rising *ε*, indicating diminished anharmonicity in polar solvents, which suppresses vibrational coupling effects.

A critical observation is the single-peak behavior of 4ICA in DMSO versus unresolved doublets in other solvents (Figure 4). This contrasts with 4CI, which displays distinct doublets across all solvents due to Fermi resonance between the C≡N stretch (bright mode) and overtone/combination bands of low-frequency indole ring vibrations (dark modes) [27]. For 4ICA, the collapse into a single peak in DMSO likely stems from strong dielectric screening, which dampens the vibrational coupling responsible for Fermi resonance. In lower-polarity solvents (e.g., THF, *ε* = 7.5), weaker screening allows partial resolution of doublets. The Fermi resonance visibility thus hinges on the balance between solvent polarity and the energy gap between coupled vibrational states. The larger dipole moment change in DMSO (Δ*μ* = 1.6731 D) further corroborates the intensified polarization that disrupts coupling.

**Figure 5:**
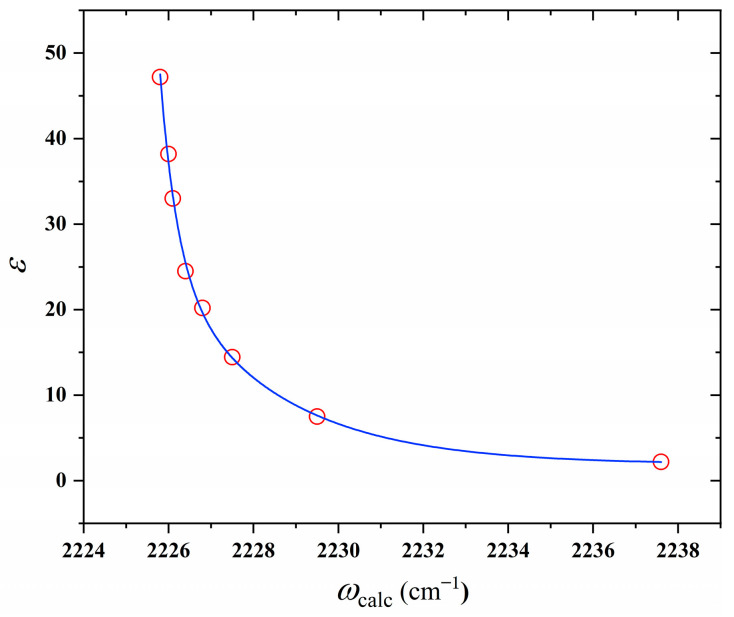
The computed NHCN vibrational frequency (*ω*_calc_) of 4ICA exhibits a correlation with the solvent dielectric constant (ε), as described by the Equation (1). The data points (red circles) obtained from various solvents are well-fitted by the equation (blue line), yielding a coefficient of determination (*R*^2^) of 0.99.

Interesting, the relationship between *ω*_calc_ and *ε* (Figure 5) is quantitatively captured by the equation:

(1) $$\varepsilon =1.90+22.8\exp\left(-\frac{\omega_{calc}-2225.79}{0.41}\right)+22.89exp(-\frac{\omega_{calc}-2225.79}{2.67})$$

This biphasic dependence indicates a sharp decline in *ε* for *ω*_calc_ > 2226 cm^−1^ (low-polarity regime) and a plateau for *ω*_calc_ < 2226 cm^−1^ (high-polarity regime). The equation reflects saturation of solvent–solute interactions beyond a critical polarity threshold, where further increases in *ε* yield negligible shifts in *ω*_calc_. This aligns with the Hughes-Ingold rule, which posits that solvent effects on reaction rates or vibrational properties diminish when charge dispersion approaches completion in highly polar environments.

In summary, the solvent dependence of 4ICA’s properties underscores the dominance of dielectric screening and specific interactions in modulating vibrational behavior. The single-peak phenomenon in DMSO arises from the suppression of Fermi resonance due to strong polarization, while the quantitative *ω*_calc_–*ε* relationship provides a predictive framework for solvent effects. Such vibrational properties of 4ICA render it an effective infrared probe, particularly suitable for studies on solvation dynamics and biomolecular microenvironments. Furthermore, when combined with the frequency-dielectric constant quantitative model, 4ICA holds promise for quantitatively mapping polarity distribution maps of biological interfaces, providing a new dimension for understanding biomolecular interactions.

### 2.4. IR Pump–Probe Spectroscopy

**Figure 6:**
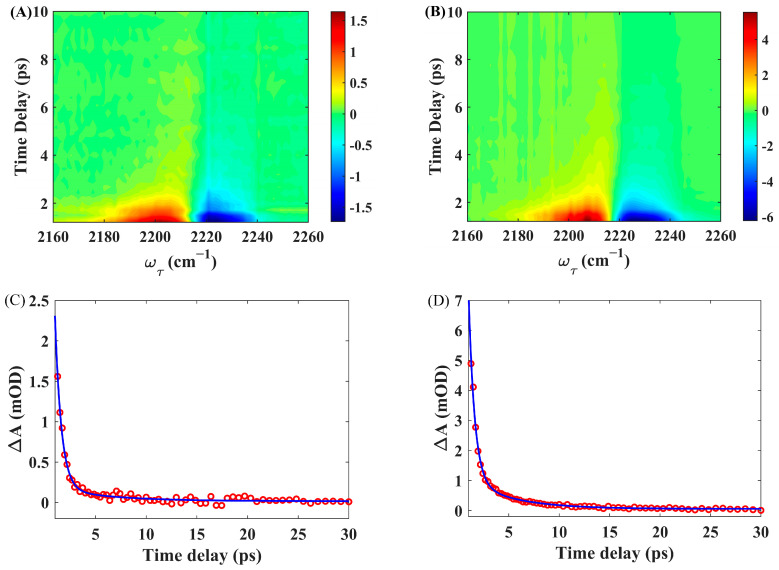
Time- and frequency-resolved transient absorption signals for 4ICA in (**A**) DMSO and (**B**) EtOH. Corresponding two-dimensional spectra as a function of probe delay time and frequency are shown for (**C**) DMSO and (**D**) EtOH. The dynamics are well described by a single-exponential decay function (blue line), with the experimental data represented by red circles.

To examine the vibrational relaxation dynamics of the NHCN stretching mode in 4ICA dissolved in solvents, the polarized IR pump–probe experiments were performed. DMSO and EtOH were selected as representative solvents. Figure 6A presents the frequency-resolved pump–probe spectra of the NHCN stretching mode of 4ICA in DMSO. Two distinct spectral features are clearly resolved: a negative band centered at approximately 2220 cm^−1^, assigned to ground-state bleach (GSB) and stimulated emission (SE), and a positive band centered near 2205 cm^−1^, attributed to excited-state absorption (ESA). The observed frequency separation between the GSB and ESA bands reflects the vibrational anharmonicity and homogeneous line broadening of the system. As expected, the signal decays to zero at longer pump–probe delays, indicating complete energy relaxation. A schematic diagram illustrating the dynamics involving GSB, SE, and ESA can be found in standard references on ultrafast vibrational spectroscopy [35,36,37].

In order to determine the vibrational lifetime (τ) of ω_(NHCN)_ mode for 4ICA in DMSO, we analyzed the temporal evolution of integrated positive peak areas at 2205 cm^−1^ in transient absorption spectra (Figure 6C). Single-exponential function fitting of the decay profile yielded *τ* = 1.35 ± 0.04 ps. Notably, this lifetime is significantly shorter than values observed for NC modes in analogous indole-based probes [30,31].

Complementary polarization-controlled infrared pump–probe measurements were performed in EtOH (Figure 6B), where the temporal decay profiles of integrated positive peaks (Figure 6D) at 2215 cm^−1^ similarly exhibited a single-exponential behavior as well. Analysis revealed a vibrational lifetime of *τ* = 1.13 ± 0.03 ps for the NHCN stretch in this solvent, which is shorter than that in DMSO. This result further supports that the DMSO environment, via a weaker Fermi resonance, not only modifies the spectral band shape of 4ICA but also considerably extends the excited-state lifetime of its NHCN stretch. Conversely, in solvents like ethanol, the environment facilitates faster energy relaxation, leading to a markedly shorter lifetime.

## 3. Materials and Methods

### 3.1. Materials and Sample Preparation

The 4ICA compound was synthesized and characterized. Solvents, sourced from Sigma-Aldrich (St. Louis, MO, USA) or J&K Scientific (Shanghai, China), were used without further purification. All experiments used freshly prepared solutions.

### 3.2. Spectroscopic Measurements

All FTIR spectra were recorded on a Bruker VERTEX 70 spectrometer (Bruker, Karlsruhe, Germany) at 22 °C with 0.5 cm^−1^ resolution. The sample solution was injected into an IR sample cell with 200 μm spacer thickness and 2 mm calcium–fluoride (CaF_2_) window thickness. The final FTIR absorption spectrum was obtained by subtracting the background spectrum. This procedure was repeated for each sample concentration, and a complete set of concentration-dependent IR absorption spectra were collected. Experimental details for the transient absorption experiment are described elsewhere [38,39]. Briefly, the transient absorption experiment was conducted using a commercialized setup (PhaseTech, Rapid City, SD, USA). A 2.5 W, 35 fs, 800 nm laser from Astrellia (Coherent, Santa Clara, CA, USA) was used to pump an OPA and DFG system (Topas Prime, Light Conversion, Vilnius, Lithuania), generating a mid-IR pulse with a central wavelength of ~2200 cm^−1^, pulse duration of 150 fs, and power of 16 mW. The mid-IR pulse was directed into the transient absorption setup and split into two parts by a beam separator: approximately 95% was transmitted as the pump pulse to excite the sample, while about 5% was reflected as the probe pulse. The pump pulse passed through a delay stage to adjust the optical path length and was then focused onto the sample, coincident with the probe pulse. After interacting with the sample, the pump pulse was blocked, and the probe pulse was guided into the MCT (Mercury-Cadmium-Telluride) detector to acquire the transient absorption signals. For such experiment, all liquid samples are encapsulated in a customized cuvette consisting of two CaF_2_ windows separated by a PTFE spacer. The thickness of the spacer is adjustable, typically kept at 30 μm, and adjusted according to the signal of the actual sample.

### 3.3. Computational

Electronic structure calculations were performed using Gaussian 16 (revision A.02) [40]. Geometries were optimized and vibrational frequencies calculated at the B3LYP/6-31+G(d,p) level. The absence of imaginary frequencies confirmed true energy minimum on the potential energy surface. Single-point energy calculations were then conducted on the optimized structures. To account for solvation effects, geometry optimization of 4ICA was executed in vacuum and with the CPCM implicit solvation model [41]. Eight solvents were studied: DMSO, DMF, MeOH, EtOH, IPA, THF, cyclopentanone and 1,4-dioxane.

## 4. Conclusions

In conclusion, we have successfully synthesized and characterized 4ICA as a highly sensitive infrared probe for detecting local environmental changes through its NHCN stretching vibration. Key findings demonstrate that 4ICA exhibits a remarkably large solvent-dependent frequency shift, strong correlation with solvatochromic parameters (π*+β), and a significantly enhanced transition dipole moment compared to classical cyanotryptophan analogs. These features make it especially promising for applications in two-dimensional IR spectroscopy and other nonlinear optical methods where signal intensity and environmental sensitivity are critical.

Theoretical calculations support experimental observations, revealing modulation of vibrational frequencies and Fermi resonance behavior by solvent polarity and specific interactions. Moreover, ultrafast pump–probe experiments illustrated distinct vibrational relaxation dynamics in protic and aprotic solvents, reinforcing the role of local solvation in vibrational energy transfer.

Given its synthetic accessibility, pronounced solvatochromism, and strong transition dipole strength, 4ICA emerges as a superior IR probe for deciphering hydration, polarity, and hydrogen-bonding networks in biomolecular systems.

## Figures and Tables

**Table 1:** Vibrational band parameters and solvent properties for 4ICA. The band center (cm^−1^) and FWHM (cm^−1^) of the NHCN stretching mode are listed alongside solvent Kamlet–Taft parameters: polarizability (π*), hydrogen bond acceptor ability (β) and hydrogen bond donor strength (α).

Solvent	*ω*_(__NHCN__)_	FWHM	π*	β	α
dimethyl sulfoxide (DMSO)	2217.5	14.0	1	0.76	0
*N*,*N*-Dimethylformamide (DMF)	2219.5	18.0	0.88	0.69	0
isopropanol (IPA)	2224.5	26.7	0.48	0.95	0.76
ethanol (EtOH)	2225.5	22.2	0.54	0.77	0.83
methanol (MeOH)	2226.0	20.5	0.6	0.62	0.93
cyclopentanone	2226.5	19.8	0.76	0.52	0
tetrahydrofuran (THF)	2228.5	20.2	0.58	0.55	0
1,4-dioxane	2230.5	20.4	0.55	0.37	0
Acetonitrile (ACN)	2231.0	21.2	0.75	0.31	0.19

**Table 2:** The transition dipole strength (in units of D^2^) of the nitrile and isonitrile for indole derivatives in various solvents.

Compound	Solvent			
	Ethanol	DMSO	DMF	MeOH
4ICA	205.0	242.5	231.5	241.3
5ICI [30]	85.3	125.2	117.3	97.5
6MICI [31]	79.8	103.4	100.1	81.5
4CI	77.6	125.4	120.9	81.1

**Table 3:** Computed Properties of 4ICA in implicit solvent environments using the CPCM.

Solvents	*E*_solvation_	*μ*	*Δμ*	*ω*_calc_	*ω*_exp_	*Δω*	*L_CN_*	*ΔL_CN_*	*f_KBM_*	*ε*
Vacuum		4.7969		2246.7			1.1685			
DMSO	−41.3466	6.4699	1.6731	2225.8	2217.5	−8.31	1.1705	0.00201	0.484	47.2
DMF	−41.0657	6.4565	1.6596	2226.0	2219.5	−6.50	1.1705	0.00199	0.481	38.2
MeOH	−40.8714	6.4471	1.6503	2226.1	2226.0	−0.13	1.1705	0.00198	0.478	33
EtOH	−40.3857	6.4242	1.6273	2226.4	2225.5	−0.95	1.1705	0.00195	0.470	24.5
IPA	−39.7976	6.3963	1.5994	2226.8	2224.5	−2.34	1.1704	0.00191	0.464	20.2
cyclopentanone	−38.7054	6.3451	1.5482	2227.5	2226.5	−1.04	1.1704	0.00184	0.450	14.45
THF	−35.6992	6.2067	1.4098	2229.5	2228.5	−1.31	1.1702	0.00166	0.406	7.5
1,4-dioxane	−21.2248	5.5861	0.7892	2237.6	2230.5	−0.95	1.1694	0.00087	0.222	2.2

## Data Availability

The data presented in this study is available on request from the first author.

## Supplementary Materials

The following supporting information can be downloaded at: https://www.mdpi.com/article/10.3390/molecules30204063/s1. Table S1. Spectral parameters of 4CI in different solvents and each solvent with its Kamlet–Taft parameters, π* (polarizability), β (hydrogen bond acceptor), α (hydrogen bond donor) and ε (dielectric constant) list in the table; Table S2. Fitting parameters of the fitting function between the probe delay time and frequency for 4ICA in DMSO and EtOH. Such model function is given by I(t) = Ae − t/τ + y_0_, where A is the amplitude, τ is the vibrational lifetime, and y_0_ is the baseline offset representing the long-term signal after complete relaxation; Figure S1. ^1^HNMR of the sample (4ICA); Figure S2. Mass Spectrometry of the sample (4ICA); Figure S3. HPLC report on the sample (4ICA); Figure S4. ω_NHCN_ versus solvent parameter π* (left) and β (right); Figure S5. ω_NHCN_ versus solvent FWHM; Figure S6. The linear relationship between Δ*μ* of 4ICA and *f*_KBM_.
