# Supplementary material for: Synthesis and Characterization of 4-Indolylcyanamide: A Potential IR Probe for Local Environment
Source: Molecules. 2025 Oct 12;30(20):4063. doi: 10.3390/molecules30204063 (PMC12566075; doi:10.3390/molecules30204063)

Supplementary Material for:

“Synthesis and Characterization of 4-Indolylcyanamide: A Potential IR Probe for Local Environment”

Table S1. Spectral parameters of 4CI in different solvents and each solvent with its Kamlet–Taft parameters, π*(polarizability), β(hydrogen bond acceptor), α(hydrogen bond donor) and ε(dielectric constant) list in the table.

Solvent	ω_1	ω_2	π*	β	α	ε
DMSO	2213.3	2229.2	1	0.76	0	47.2
DMF	2213.8	2229.2	0.88	0.69	0	38.2
EtOH	2216.9	2233.1	0.48	0.95	0.76	20.2
MeOH	2217.2	2233.3	0.6	0.62	0.93	33
1,4-dioxane	2216.1	2230.5	0.55	0.37	0	2.2

Table S2. Fitting parameters of the fitting function between the probe delay time and frequency for 4ICA in DMSO and EtOH. Such model function is given by $I(t)=Ae^{-t/\tau}+y_0$, where A is the amplitude, τ is the vibrational lifetime, and y_0 is the baseline offset representing the long-term signal after complete relaxation.

Solvent	A	τ	y_0
DMSO	2.07	1.35	0.02
EtOH	1.41	1.13	0.02

Figure S1. ^{1}HNMR of the sample (4ICA).

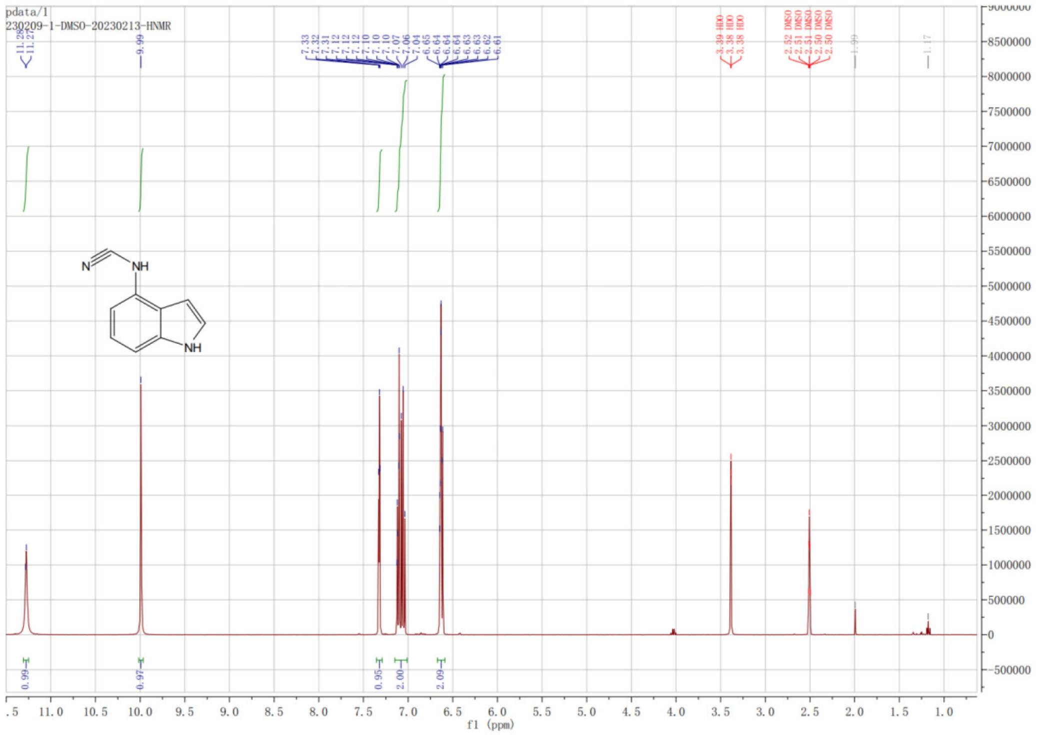

Figure S2. Mass Spectrometry of the sample (4ICA)

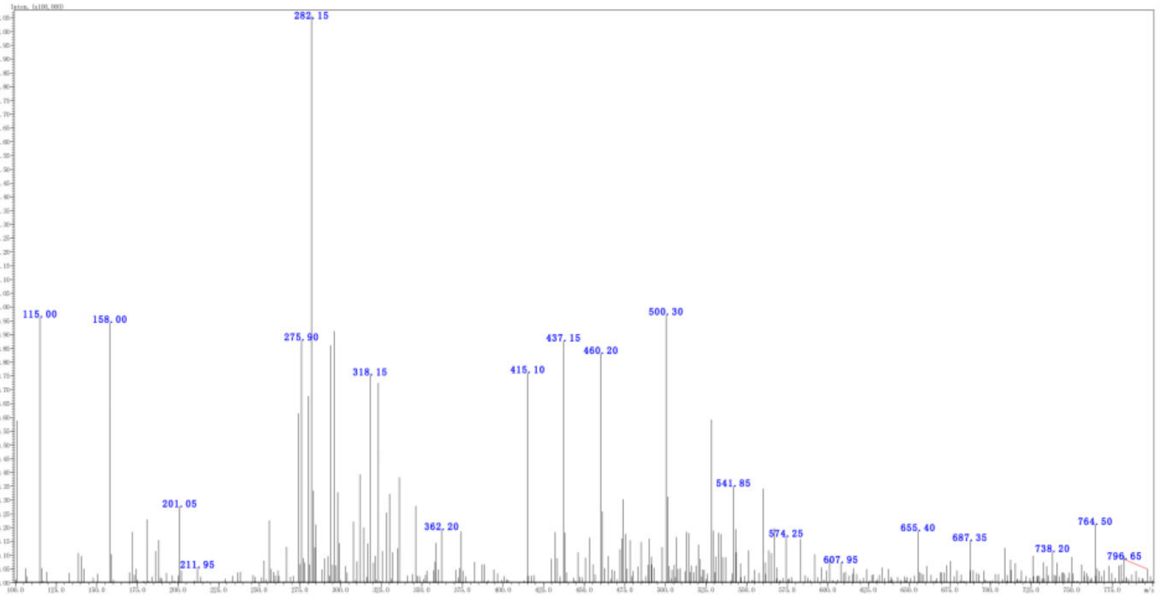

Figure S3. HPLC report on the sample (4ICA)

HPLC Report

Sample Information

Customer Code: KSVP Code:KP2700

Lot No.:20230215

Phase A: 0.1% TFA in deionized water

Phase B: 0.08% TFA in acetonitrile

Flow rate:1.0mL/min

Wavelength:214nm

Analytical column type:YMC-Pack ODS-A、120A、5um、4.6*250mm

Dissolution method:25%ACN+75%H2O

Inj. Volume:2uL

Date Acquired:20230217,12:44:04

Gradient：

Time（min）	Phase A	Phase B
0.01	99%	1%
2.00	99%	1%
22.00	1%	99%
30.00	Stop	

Chromatogram

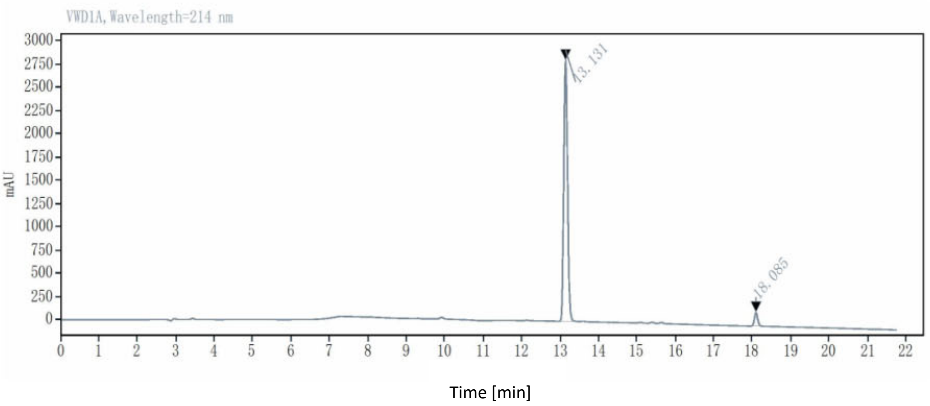

Peak	Ret time	Area	Area%	Height	Height%
1	13. 131	19421. 3464	95. 82	2807. 926	95. 14
2	18. 085	846. 8135	4. 18	143. 589	4. 86

Figure S4. ω_{NHCN} versus solvent parameter π*(left) and β (right).

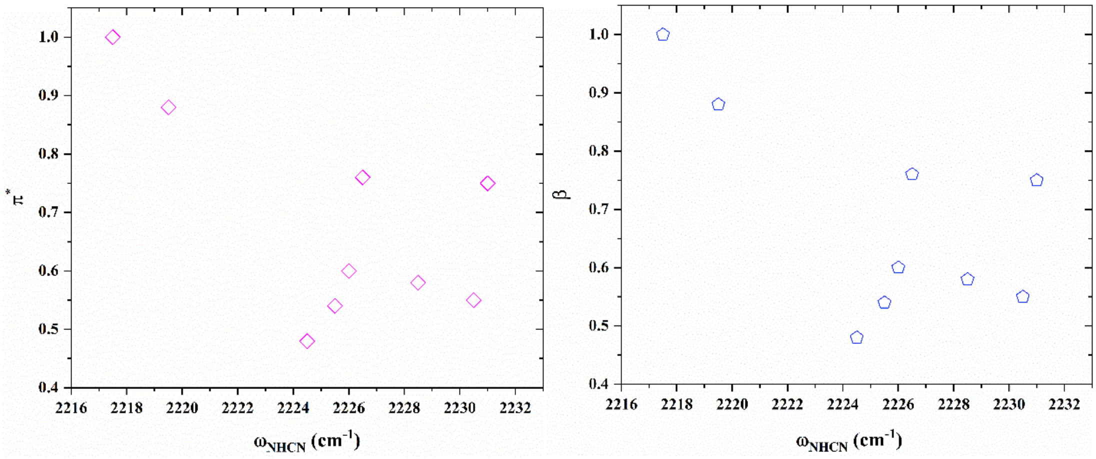

Figure S5. ω_{NHCN} versus solvent FWHM.

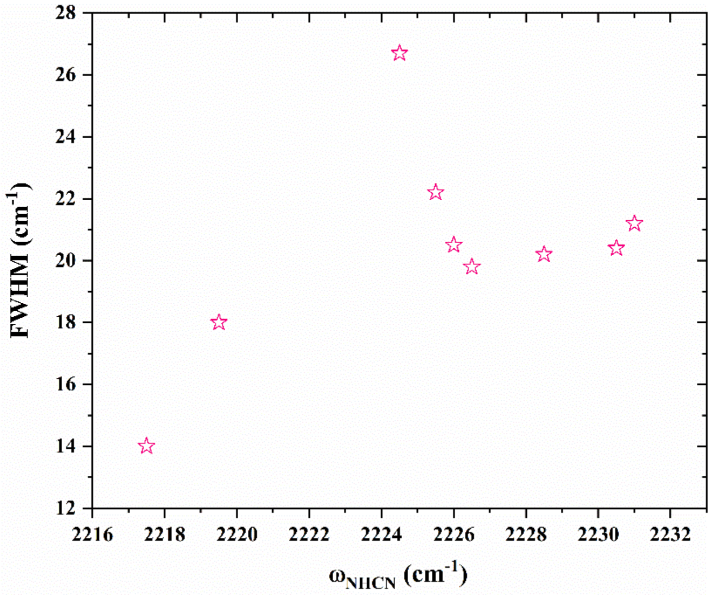

Figure S6. The linear relationship between $\Delta\mu$ of 4ICA and f_{KBM}

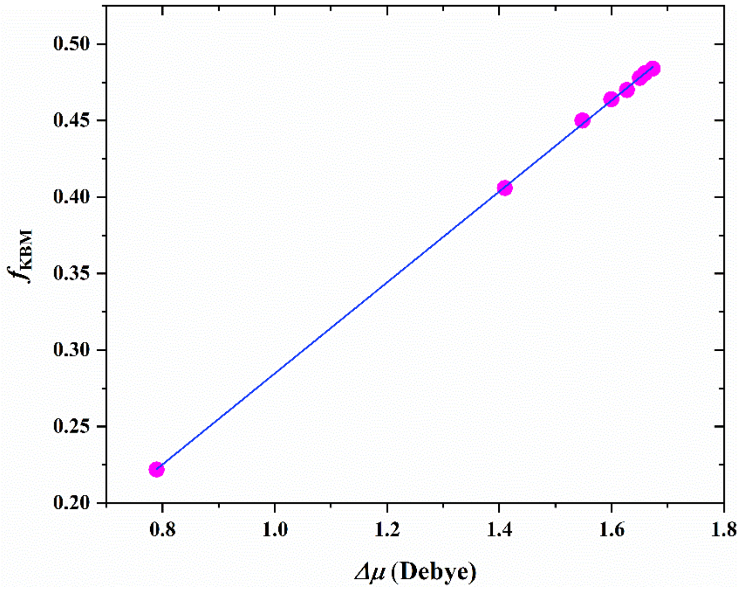

Supplement: Supplementary file 1 [file molecules-30-04063-s001.zip › molecules-3862108-supplementary.pdf]
